# Genome Annotation Provides Insight into Carbon Monoxide and Hydrogen Metabolism in *Rubrivivax gelatinosus*

**DOI:** 10.1371/journal.pone.0114551

**Published:** 2014-12-05

**Authors:** Karen Wawrousek, Scott Noble, Jonas Korlach, Jin Chen, Carrie Eckert, Jianping Yu, Pin-Ching Maness

**Affiliations:** 1 Department of Chemical and Petroleum Engineering, University of Wyoming, Laramie, Wyoming, United States of America; 2 Biosciences Center, National Renewable Energy Laboratory, Golden, Colorado, United States of America; 3 Pacific Biosciences, Menlo Park, California, United States of America; 4 Department of Energy Plant Research Laboratory, Michigan State University, East Lansing, Michigan, United States of America; Louisiana State University and A & M College, United States of America

## Abstract

We report here the sequencing and analysis of the genome of the purple non-sulfur photosynthetic bacterium *Rubrivivax gelatinosus* CBS. This microbe is a model for studies of its carboxydotrophic life style under anaerobic condition, based on its ability to utilize carbon monoxide (CO) as the sole carbon substrate and water as the electron acceptor, yielding CO_2_ and H_2_ as the end products. The CO-oxidation reaction is known to be catalyzed by two enzyme complexes, the CO dehydrogenase and hydrogenase. As expected, analysis of the genome of *Rx. gelatinosus* CBS reveals the presence of genes encoding both enzyme complexes. The CO-oxidation reaction is CO-inducible, which is consistent with the presence of two putative CO-sensing transcription factors in its genome. Genome analysis also reveals the presence of two additional hydrogenases, an uptake hydrogenase that liberates the electrons in H_2_ in support of cell growth, and a regulatory hydrogenase that senses H_2_ and relays the signal to a two-component system that ultimately controls synthesis of the uptake hydrogenase. The genome also contains two sets of hydrogenase maturation genes which are known to assemble the catalytic metallocluster of the hydrogenase NiFe active site. Collectively, the genome sequence and analysis information reveals the blueprint of an intricate network of signal transduction pathways and its underlying regulation that enables *Rx. gelatinosus* CBS to thrive on CO or H_2_ in support of cell growth.

## Introduction

*Rubrivivax gelatinosus* CBS was originally isolated from soil in Denver, Colorado. It is a purple non-sulfur (PNS) photosynthetic bacterium belonging to the family of *Rhodospirillaceae* [1]. Similar to most PNS in this family, *Rx. gelatinosus* is versatile with various modes of growth and energy metabolism. It carries out anoxygenic photosynthesis using electrons derived from organic acids and energy from sunlight, aerobic respiration using organic acids, and N_2_ fixation to ammonium in support of cell growth [2], [3]. Moreover, upon exposure to carbon monoxide (CO) in the culture gas phase, two *Rx. gelatinosus* strains, i.e., CBS and S1 can both carry out a water-gas shift reaction according to the equation CO + H_2_O → CO_2_ + H_2_ [4]-[7]. The CO_2_ and H_2_ products are then assimilated into new cell mass either in the light or in darkness, both under anaerobic condition. The latter growth mode in darkness can use CO as the sole carbon and energy source, hence coupling CO oxidation to energy conservation by a proton/sodium gradient generated by electron transfer via the CO-linked hydrogenase [8], [9].

The CO metabolic pathway in the PNS *Rhodospirillum rubrum* is well characterized and has served as the model system to unravel the genes and enzymes involved in CO metabolism. Upon exposure to CO, two *coo* (CO oxidation) operons are induced, with the *cooFSCTJ* operon encoding CO dehydrogenase (CODH) and related Ni-insertion proteins [10], [11], and the *cooMKLXUH* operon encoding a NiFe-hydrogenase [12], [13]. Transcription of both operons is under the control of the heme-containing CO-sensing transcription factor *cooA* [14]. The CooF protein contains iron-sulfur (FeS) clusters which likely mediates electron transfer between CODH and the CO-linked CooMKLXUH hydrogenase [15], [16]. Prior research in *Rx. gelatinosus* CBS has identified a *cooMKLXUH* operon encoding a CO-inducible hydrogenase [17]. This hydrogenase is likely a hexameric protein with high degree of amino acid identity to its homologs in *Rs. rubrum*, *Carboxydothermus hydrogenoformans* [18], *Methanosarcina barkeri* [19], and *Thermoanaerobacter tengcongensis* [20]. This type of hexameric NiFe-hydrogenases shares a common feature in that the hydrophilic subunits CooLXUH display sequence similarity to the energy-conserving NADH: quinone oxidoreductase (complex I). This class of hydrogenases is classified as the Group 4 energy-converting hydrogenase (Ech) [21], consistent with its role in proton-pumping reaction to yield energy from CO oxidation and H_2_ production [9], [22]. The CO-dependent H_2_ production hence has important ramifications in microbial energy generation during C1 carbon metabolism.

Although the genomes of the *Rx. gelatinosus* strains ATCC17011 and IL144 have been sequenced, neither contains homologs of the *cooFSCTJ* or the *cooMKLXUH* genes [23]. When tested, the strain ATCC17011 failed to metabolize CO (NREL, unpublished work). As such the versatility of energy metabolism in *Rx. gelatinosus* CBS especially regarding CO metabolism and CO-linked H_2_ production prompted us to sequence its genome [24], which is the first sequenced genome for a *Rx gelatinosus* strain capable of metabolizing CO.

Detailed genome annotation revealed a *cooFSC* and *cooMKLXUH* gene cluster likely encoding the protein machinery responsible for CO oxidation and H_2_ production. We uncovered a set of hydrogenase maturation genes (*hypABFCDE*), which is clustered near the *coo* operon and presumably assembles the active site of the Ech hydrogenase [25], [26]. Moreover, we uncovered a H_2_-uptake hydrogenase and a H_2_-sensing hydrogenase along with a second set of *hyp* maturation genes, with a genome arrangement similar to that in *Ralstonia eutropha* [27]. The *Rx. gelatinosus* CBS genome hence provides the blueprint to an intricate signal transduction network governing H_2_ and CO sensing, regulation, and metabolism.

## Materials and Methods

### Genome Sequencing

SMRTbell template libraries were prepared as previously described [28]. Two different sized SMRTbell template libraries were employed. Genomic DNA samples were either sheared to an average size of ∼800 base pairs via adaptive focused acoustics (Covaris; Woburn, MA, USA) or to a target size of approximately 8–10 kilobase pairs using Covaris g-TUBEs (Woburn, MA, USA). Fragmented DNA was then end repaired and ligated to hairpin adapters. Incompletely formed SMRTbell templates were digested with a combination of Exonuclease III (New England Biolabs; Ipswich, MA, USA) and Exonuclease VII (Affymetrix; Cleveland, OH, USA). SMRT Sequencing was carried out on the Pacific Biosciences *RS* (Menlo Park, CA, USA) using C2 chemistry with standard protocols for either small or large insert SMRTbell template libraries.

### Genome Assembly

The *de novo* genome assembly was performed using hybrid assembly protocols, which is based on error correction of the long-insert library of SMRT sequencing reads with the short-insert library, circular consensus sequencing (CCS) SMRT sequencing reads [29], [30]. The algorithm is available in SMRT Analysis version 1.3.3. The initial assembly resulted in three contigs with sizes of 4,724,250, 362,142 and 241,393 bases. The 241 kb contig was determined to be a distinct genomic element without any sequence similarity to the other two contigs, and was concluded to be circularly closed due to overlapping ends from the assembler (Figure S1). The remaining two contigs were found to have a region of high sequence similarity near the end of the 4.7 Mb and the very beginning of the 362 kb contig. It overlaps with a coverage anomaly at the end of the 4.7 Mb contig, indicating the presence of a 47 kb high-copy phage/plasmid element that has sequence identity with this region in the chromosome over a stretch of ∼43 kb (Figure S2). The 47 kb high-copy element was represented by a new contig, and the 4.7 Mb and 362 kb contigs were connected to yield the full-length, 5.1 Mb bacterial chromosome. The chromosome had overlapping, self-similar ends, indicating that it is circularly closed. Such end-overlaps are typical for *de novo* assemblies on circularly closed genomic elements, and were trimmed manually. Approximately 93% of the long-insert library reads mapped back to the *de novo* assembly which is typical for SMRT sequencing.

We also applied a more recent hierarchical genome assembly algorithm (HGAp) on the long-insert library data only, and obtained identical results for the final genome assembly. HGAp is available at www.pacbiodevnet.com/hgap. Dot plots were generated using Gepard [31]. For the quality control of remapping reads back to the assembly, reads were mapped using the BLASR mapper (http://www.pacbiodevnet.com/SMRT-Analysis/Algorithms/BLASR) and the Pacific Biosciences' SMRT Analysis pipeline (http://www.pacbiodevnet.com/SMRT-Analysis/Software/SMRT-Pipe) using the standard mapping protocol.

## Results and Discussion

### Overview of Subsystem Category

**Figure 1:**
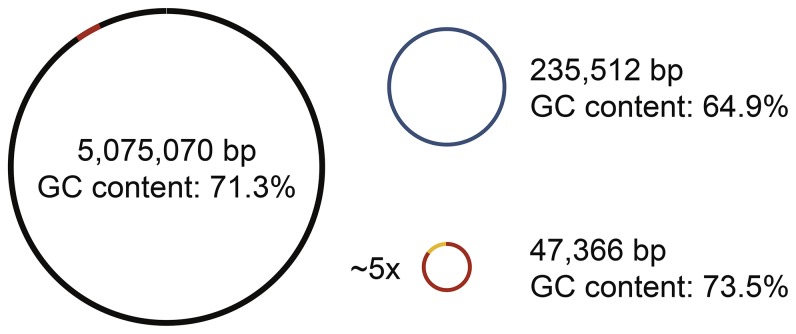
*De novo* genome assembly results for *Rubrivivax gelatinosus* CBS, resulting in one bacterial chromosome and two satellite DNA elements. The common section of the small satellite and the chromosome are highlighted in red. Not drawn to scale.

**Figure 2:**
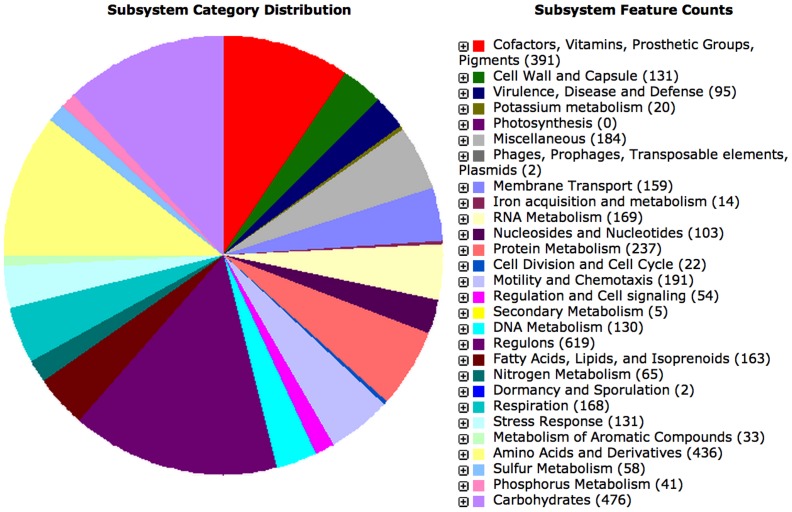
Organism Overview for *Rubrivivax gelatinosus* CBS. There are 643 subsystems, 4852 coding sequences, and 58 RNAs.

Hybrid, *de novo* genome assembly from long-insert library SMRT sequencing reads and short-insert library CCS SMRT sequencing reads resulted in three contigs representing one circularly closed bacterial chromosome with a size of 5,075,070 bases and a GC content of 71.3%, a 235,5122 basepair plasmid with a GC content of 64.9%, and a 47,366 basepair plasmid with a 73.5% GC content (Figure 1). In order to annotate the *Rx. gelatinosus* CBS genome, we submitted all contigs to RAST (Rapid Annotation using Subsystem Technology), a fully-automated service for annotating bacterial and archaeal genomes [32]. With RAST, we discovered 4,910 genes in *Rx. gelatinosus* CBS. The subsystem distribution in the genome shown in Figure 2 reveals the majority of genes encode regulons and genes involved in metabolism of carbohydrates, amino acids and derivatives. Specifically, we found 41 predicted hydrogenase genes and 158 predicted dehydrogenase genes in the genome, including 6 new hydrogenases genes and 5 new dehydrogenases genes that have not been reported in [24].

### CO Metabolism via CO Dehydrogenase

**Figure 3:**
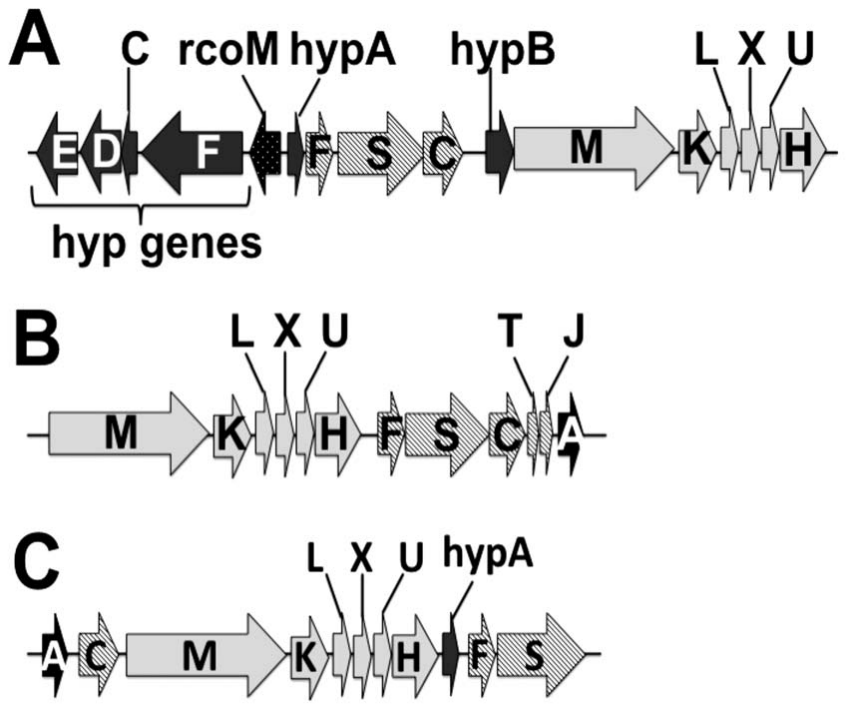
Organization of genes encoding anaerobic CO metabolism in multiple bacteria. (A) Gene structure of CO metabolism genes in *Rubrivivax gelatinosus* CBS. The *hyp* genes putatively responsible for *coo* hydrogenase maturation are clustered among the *coo* CODH and hydrogenase genes. The gene encoding RcoM, the putative transcription factor for the *coo* CODH and hydrogenase genes is located among the *hyp* genes within this cluster of CO metabolism genes. (B) Gene structure of CO metabolism genes in *Rhodospirillum rubrum*. The *cooA* gene, encoding the CooA CO-responsive transcription factor, directly follows the CODH genes. (C) Gene structure of CO metabolism genes in *Carboxydothermus hydrogenoformans*. *C. hydrogenoformans* has five CODH complexes, but the genes for only one CODH are co-located with the genes for an energy-conserving hydrogenase [36]. The *cooA* and *hypC* genes precede the *cooMKLXUH* hydrogenase genes, which are followed by *hypA* and the *cooFS* CODH genes.

Genome annotation in *Rx. gelatinosus* CBS identified *cooFCS* genes (Figure 3), which display high levels of homology with their counterparts in *Rs. rubrum*, *C. hydrogenoformans*, and *D. vulgaris* str. Hildenborough (Table 1). These genes are presumably responsible in *Rx. gelatinosus* CBS for CO oxidation catalyzed by CODH (encoded by *cooS*), followed by electron transfer to CooF, an FeS protein. Critical residues for the Ni-Fe-S active site in CooS are highly conserved [33], and in CBS these conserved residues are H266, C293, G457, C458, C489, C540, T581, and K583. CooS is predicted to have one FeS cluster in the N-terminal half of the protein coordinated by conserved cysteine residues at C48, C51, C56, and C70. The CooF protein in *Rs. rubrum* contains conserved cysteine motifs to coordinate up to four FeS clusters [15], [16], while the CBS CooF protein sequence predicts only three FeS clusters. However, EPR data of *Rs. rubrum* CooF revealed that there are likely only two FeS clusters present per CooF monomer [16]. CooF likely mediates electron transfer from the CooS active site to the Coo hydrogenase (discussed below).

**Table 1:** Similarity and identity of select CO dehydrogenase proteins and CO-responsive transcription factors between *Rubrivivax gelatinosus* CBS with other bacterial species, generated by EMBOSS 6.3.1: matcher search [62].

Protein and organism	Similarity%	Identity%
		
**CooF**		
*Rs. rubrum*	71	60
*C. hydrogenoformans*	63	49
* D. vulgaris*	65	48
**CooS**		
*Rs. rubrum*	76	61
*C. hydrogenoformans*	77	59
* D. vulgaris*	60	44
**CooC**		
*Rs. rubrum*	70	52
*C. hydrogenoformans*	65	46
*D. vulgaris*	58	46
**RcoM**		
*B. xenovorans* RcoM-1	52	32
*B. xenovorans* RcoM-2	58	35
*Rs. rubrum*	43	28
**CooA**		
*Rx. gelatinosus* IL144	100	98
*Rx. benzoatilyticus* JA2	95	90
*Rs. rubrum*	57	36
*C. hydrogenoformans*	61	37
**CowN**		
*Rx. gelatinosus* IL144	100	99
*Rx. benzoatilyticus* JA2	92	91
*Rs. rubrum*	50	31
**CoxS**		
*Rx. gelatinosus* IL144	100	100
*O. carboxidovorans*	74	58
**CoxL**		
*Rx. gelatinosus* IL144	99	99
*O. carboxidovorans*	54	38
**CoxM**		
*Rx. gelatinosus* IL144	100	100
*O. carboxidovorans*	58	43
**CoxD**		
*Rx. gelatinosus* IL144	100	99
*O. carboxidovorans*	64	48

Strain Hildenborough was chosen for the comparison of *Desulfovibrio gigas*. Since *Rx. benzoatilyticus* does not contain the *cox* genes for aerobic CO oxidation, only predicted protein sequences from *Rx. gelatinosus* IL144 and *Oligotropha carboxidovorans* were used for comparison with *Rx. gelatinosus* CBS.

Similar to other complex metalloproteins, the CODH enzyme requires maturation factors, or chaperones, for the correct insertion of metals to form the holoprotein. In *Rs. rubrum*, it was found that CooCTJ are involved in the insertion of Ni into the CooS protein [10]. CooC is a membrane-associated protein that is believed to couple ATP hydrolysis with Ni insertion into the CODH active site [34]. There is less Ni insertion into the active site of CooS in either *cooT* or *cooJ* mutants, but there was virtually no CODH activity in a *cooC* mutant [34], [35]. This implies that CooC is the most important of these proteins for Ni insertion. A CooC homolog is evident in *Rx. gelatinosus* CBS, but the genes encoding CooT and J are not present (Figure 3), similar to *C. hydrogenoformans*. Yet even without clear homologs of *cooT* and *cooJ*, both *Rx. gelatinosus* CBS and *C. hydrogenoformans* produces an active CODH, as evidenced by growth on CO as a sole carbon source [1], [36]. This suggests that either CooC alone may suffice for Ni insertion into the CooS active site in the latter microbes, or Ni insertion is assisted by some yet to be identified proteins.

In addition to the *coo* genes for anaerobic CO oxidation, *Rx. gelatinosus* CBS was found to also have the *coxSLMD* genes for aerobic CO oxidation. Aerobic CO oxidation has been best studied in *Oligotropha carboxidovorans*, and in this organism *coxSLM* encode the carbon monoxide dehydrogenase enzyme. CoxS is the small subunit and contains 2 FeS clusters. CoxM, the medium-sized subunit, contains an FAD binding site, and the enzymatic CoxL is the large, catalytic subunit and contains the Mo-Cu active site. The CoxD protein is involved in biosynthesis of the active site metallocluster [37]. While *Rx. benzoatilyticus* does not contain any of the *coxSLMD* genes, the *Rx. gelatinosus* IL144 genome does. The IL144 strain is similar to *Rx. gelatinosus* CBS in genome size (around 5 Mb) and GC content (around 71%), as well as numbers of predicted genes (close to 5000) [23]. As shown in Table 1, the *Rx. gelatinosus* IL144 CoxSLMD predicted proteins share 99–100% sequence identity with those predicted in *Rx. gelatinosus* CBS. While the genes encoding *coxSLMD* have been identified in both the *Rx. gelatinosus* CBS and *Rx. gelatinosus* IL144 genomes, no expression data exists for these genes in either organism and the functionality of an aerobic mode of CO metabolism has not been confirmed in either strain.

### Transcription Regulation of CO Metabolism

Anaerobic carbon monoxide metabolism in both *Rs. rubrum* and *Rx. gelatinosus* CBS is regulated by CO. In *Rs. rubrum*, CO induces increased expression of the genes encoding CODH and hydrogenase [12], [38], [39]. CooA is the CO-responsive transcription factor that regulates expression of *coo* genes in *Rs. rubrum* [12], [14], [40], [41]. The CooA protein contains an N-terminal b-type heme for gas sensing and a C-terminal helix-turn-helix motif to bind DNA [42]. CO metabolism in *Rx. gelatinosus* CBS is also regulated by the presence of CO with CO-dependent growth and the appearance of Coo hydrogenase proteins CooH and CooL strictly depending on the presence of CO [1], [17]. Genome annotation revealed a *cooA* gene in *Rx. gelatinosus* CBS, yet it is not clustered with the *cooFSC* genes as in *Rs rubrum*. CooA in *Rx. gelatinosus* CBS is only 36% identical and 57% similar to the well-studied CooA in *Rs. rubrum* (Table 1). As expected based on protein alignment, the CooA in *Rx. gelatinosus* CBS is most similar to its counterpart in *Rx. gelatinosus* IL144 *and Rx. benzoatilyticus* (Table 1). However, neither of the latter two microbes metabolizes CO anaerobically and genes encoding the CODH and Coo hydrogenase necessary for anaerobic CO metabolism are not present [23].

Interestingly, the *Rx. gelatinosus* CBS *cooA* gene is adjacent to the *cowN* gene in the genome. In *Rs. rubrum*, the CowN protein is responsible for the protection of nitrogenase from inactivation by CO [43]. While it is known that *Rx. gelatinosus* CBS can fix nitrogen while consuming CO as the sole carbon source [1], the mechanism of the protection of nitrogenase is unknown. The *Rx. gelatinosus* CBS, *Rx. gelatinosus* IL144, and *Rx. benzoatilyticus* genomes do all contain the *cowN* gene, with protein sequence identities ranging from 91-99% with the predicted *Rx. gelatinosus* CBS CowN protein (Table 1). Since neither *Rx. gelatinosus* IL144 nor *Rx. benzoatilyticus* contain genes for the anaerobic metabolism of CO, the role of CooA and CowN proteins is unclear if these genes are indeed expressed. It is worth noting that the genome arrangement in *Rx. gelatinosus* CBS is the reverse of what is observed in *Rs. rubrum*, where *cooA* is clustered with coo genes and the gene encoding the RcoM transcription factor (described below) is adjacent to the *cowN* gene [43], suggesting that *cooA* may regulate *cowN* expression in *Rx. gelatinosus* CBS.

*Rx. gelatinosus* CBS harbors a single copy of the *rcoM* gene, which is clustered with the *coo* genes responsible for anaerobic CO oxidation (Figure 3). RcoM (Regulator of CO Metabolism) was identified in 2008 and is expected to regulate genes responsible for CO metabolism in *Geobacter* spp. and *Pelobacter carbinolicus* DSM 2380, since these microbes do not harbor a CooA homolog [44]. Like CooA, RcoM is a single-component transcription factor; with an N-terminal PAS sensor domain presumably used to sense CO and a C-terminal domain containing a LytTR DNA-binding domain [44], [45]. The *Rx. gelatinosus* CBS RcoM protein is predicted to contain these conserved motifs [46] and is similar to RcoM in other organisms (Table 1). Therefore, based on the location of the *rcoM* gene in the genome along with evidence that *rcoM* is absent in strains of *Rubrivivax* that do not oxidize CO, we predict that RcoM regulates anaerobic CO metabolism genes in *Rx. gelatinosus CBS.*

### Hydrogen Metabolism and Multiple Hydrogenases

#### CO-linked Hydrogenase

The *Rx. gelatinosus* CBS genome contains a *cooMKLXUH* operon (Figure 3), with genes displaying high levels of homology with their counterparts in *Rs. rubrum* and *C. hydrogenoformans* [17]. These genes encode a membrane anchored hexameric NiFe hydrogenase [47] that is responsible in *Rx. gelatinosus* CBS for CO-linked H_2_ production [7]. This operon was initially identified via transposon mutagenesis; a *cooH* mutant strain completely lost CO-linked H_2_ production [17]. The CooH subunit harbors the NiFe active site; CooL contains an FeS cluster predicted to serve as an electron relay to/from the active site in the CooH subunit; CooX also harbors FeS clusters for electron relay based on a study on its counterpart in *Methanosarcina barkeri* [17], [48]. CooU is predicted to be a soluble protein of 180 amino acids (20 kDa). Its amino acid sequence shows 39% identity and 56% similarity to CooU (annotated as NADH dehydrogenase subunit) in *Rs. rubrum*, and 34% identity and 57% similarity to CooU in *C. hydrogenoformans*. However, the specific role of CooU in the CooMKLXUH hydrogenase has yet to be determined. Gene expression from this operon is regulated by CO, as discussed above. Not only is hydrogenase activity in *Rx. gelatinosus* CBS induced by CO, so is gene transcription (data not shown) and accumulation of subunits CooL and CooH [17].

The CooM and CooK hydrogenase subunits are membrane-associated proteins. CooM is predicted to be a large membrane protein of 1255 amino acids (130 kDa) with 29–33 transmembrane segments, as predicted by DAS (http://www.sbc.su.se/~miklos/DAS/). The amino acid sequence shows 52% identity and 66% similarity to *Rs. rubrum* CooM (annotated as NADH dehydrogenase) and 46% identity and 62% similarity to *C. hydrogenoformans* CooM (annotated as carbon monoxide-induced hydrogenase, membrane anchor subunit). CooK is predicted to be a membrane protein of 319 amino acids (34 kDa), with 8 transmembrane segments predicted by DAS. Its amino acid sequence exhibits 57% identity and 74% similarity to CooK in *Rs. rubrum* (annotated as membrane bound hydrogenase subunit, MbhM), and 50% identity and 66% similarity to CooK in *C. hydrogenoformans*. Besides serving as a membrane anchor for the hydrogenase, CooM and CooK may be involved in energy generation from the CO oxidation-H_2_ production pathway catalyzed by Ech hydrogenase including that in *Rx. gelatinosus* CBS [9], [47].

The *cooMKLXUH* operon is absent from the genome of *Rx. gelatinosus* IL144 [23]. Comparison of the two *Rx. gelatinosus* genomes shows that this operon may have been present in a common ancestor and subsequently lost in the IL144 strain. This is evidenced by the observation that genes flanking the upstream and downstream of the operon are present in both microbes, including a 48-bp region identical to the 3′ of *hypE* in *Rx. gelatinosus* CBS (Figure S3).

#### H_2_-Uptake and Sensor Hydrogenases

Membrane-bound hydrogenases (MBH) are a class of NiFe hydrogenases that couple H_2_ oxidation to the respiratory chain for energy generation, hence allowing organisms to utilize H_2_ as an electron and energy source [21]. The extensively studied MBH of *R. eutropha* H16 serves as the model enzyme for comparison [49], [50]. The MBH operon of *R. eutropha* H16 contains 22 genes, including genes encoding the MBH structural subunits (*hoxKG*) and a regulatory or sensor hydrogenase (*hoxBC*), as well as associated signaling factors (*hoxJ* and *hoxA*). HoxBC (homologs of HupUV in other organisms) functions with a two-component system composed of a histidine kinase (HoxJ/HupT) and a DNA-binding response regulator (HoxA/HupR) which regulate MBH operon expression in the presence of H_2_ [51]–[53]. Analysis of the *Rx. gelatinosus* CBS genomic sequence reveals a 17,891 basepair MBH operon with 20 genes. Comparison at the nucleotide level reveals that the MBH operon in *Rx. gelatinosus* CBS displays 98% identity with the MBH operon in *Rx. gelatinosus* IL144 spanning 99% query coverage, yet shows only 80% nucleotide identity with the MBH operon in *R. eutropha* spanning 20% query coverage. Therefore, we adopted the *Rx. gelatinosus* IL144 “*hup*” gene nomenclature for the genes in the *Rx. gelatinosus* CBS MBH operon. Nevertheless, high amino acid conservation is observed between *Rx. gelatinosus* CBS, *Rx. gelatinosus* IL144, and *R. eutropha* H16 as to the respective MBH small subunit (HupA/HoxK), large subunit (HupB/HoxG), and the sensor hydrogenase small subunit (HupU/HoxB) and large subunit (HupV/HoxC) (Table 2), with their expression likely under the influence of H_2_ [51]–[53].

**Table 2:** Similarity, identity, and coverage of select hydrogenase proteins comparing the *Rubrivivax gelatinosus* CBS sensor and uptake hydrogenases to other bacterial species including *Ralstonia eutropha* H16.

		Similarity%	Identity%	Coverage%
**Sensor Hydrogenase**				
HupU	*Rx. gelatinosus* IL144	99	98	100
	*R. eutropha* H16 (HoxB)	84	74	98
HupV	*Rx. gelatinosus* IL144	99	98	100
	*R. eutropha* H16 (HoxC)	75	66	100
**Uptake Hydrogenase**				
HupA	*Rx. gelatinosus* IL144	100	100	100
	*R. eutropha* H16 (HoxK)	96	89	87
HupB	*Rx. gelatinosus* IL144	99	99	100
	*R. eutropha* H16 (HoxG)	92	86	100

HupUV is a sensor hydrogenase, with HupU being the small subunit and HupV being the large, catalytic subunit. HupAB comprises a membrane-bound uptake hydrogenase with a small HupA subunit and a large, catalytic HupB subunit. Analysis was done using a NCBI P-BLAST search.

Examining the sequence of the *Rx. gelatinosus* CBS MBH small subunit HupA reveals that it is likely an O_2_-tolerant hydrogenase. It contains two conserved supernumerary cysteines (Cys98 and Cys197) that align with the characterized Cys19 and Cys120 in the O_2_-tolerant NiFe-hydrogenases of *R. eutropha* H16 MBH [54] and *E. coli* Hyd-1 [55], whereas the O_2_-sensitive hydrogenase contain glycine residues instead. *Rx. gelatinosus* CBS contains HupI (HoxR homolog in *R. eutropha* H16), a rubredoxin-type FeS protein deemed essential in assembling the O_2_-tolerant MBH in *R. eutropha* H16 when cultured in aerobic environment [54], [56]. Future studies may reveal whether the supernumerary cysteines contribute to the O_2_-tolerance of the MBH in *Rx. gelatinosus* CBS [7].

#### NiFe Hydrogenase Maturation Factors

Genome sequencing also reveals the presence of two copies of the pluripotent *hyp* hydrogenase maturation genes. One copy (hereafter *hyp1FCDEAB*) clusters near the *coo* operon (Figure 3) and another (hereafter *hyp2ABFCDE*) is located in the MBH operon. The respective proteins from *hyp1* and *hyp2* share identities ranging from 35% to 54% (Table 3). However, *Rx. gelatinosus* CBS Hyp2 proteins display much higher identity (ranging from 60 to 77%) with the respective counterparts in the *R. eutropha* H16 MBH operon (Table 3). It is therefore likely that the *hyp1* cluster is responsible for assembling the CO-linked, H_2_-evolving hydrogenase while the *hyp2* cluster assembles the MBH in *Rx. gelatinosus* CBS. Genetic knockout of *hyp1* and/or *hyp2* will directly test this hypothesis. No *hyp* genes were found near the *coo* operon in the *Rs. rubrum* sequenced genome (Figure 3). Two sets of *hyp* maturation genes were found in the *Rs. rubrum* genome, with one partial set (*hypABC*) clustered near its MBH (HupAB) and a second set (*hypFCDEB*) clustered near a ferredoxin hydrogenase. The latter is likely a fermentative hydrogenase linking H_2_ production to formate oxidation [57] since a nearby operon contains genes encoding formate dehydrogenase, a formate transporter, and a chaperon protein for the biosynthesis of molybdenum cofactor. A second *hypA* was found in a distant location in the genome. *Rs. rubrum* therefore might recruit these *hyp* genes for maturation of its CO-linked hydrogenase.

**Table 3:** Identity percentage of *Rubrivivax gelatinosus* CBS Hyp1, Hyp2 proteins and the Hyp proteins from *Ralstonia eutropha* H16, generated by NCBI P-BLAST search.

	*Rx. gelatinosus* Hyp1 vs. Hyp2%	*Rx. gelatinosus* Hyp1 vs. *R. eutropha* Hyp %	*Rx. gelatinosus* Hyp2 vs. *R. eutropha* Hyp %
HypA	35	31	66
HypB	54	52	67
HypC	39	38	60
HypD	51	47	77
HypE	54	54	74
HypF	37	38	60

#### Physiological Relevance of CO and H_2_ Metabolism

Genetic elements and gene arrangements controlling the CO-dependent H_2_ evolution system are very similar between *Rx. gelatinosus* CBS and *Rs. rubrum*. It is therefore logical to compare the physiological relevance of the two strains regarding CO and H_2_ metabolism. CODH was first purified from *Rs. rubrum* without adding CO for its induction [38], although adding CO does increase its CODH activity significantly and CO is required for the CO-induced hydrogenase activity [58]. To the contrary, in *Rx. gelatinosus* CBS the presence of CO during growth is required to afford CODH activity linking CO oxidation to the reduction of methyl viologen [7]. The CO-inducible hydrogenase activity in *Rs. rubrum* is reported to be insensitive to CO, retaining 40% activity in 100% CO gas phase (880 µM) [39]. However, the chromatophore membranes used for the above hydrogenase assay also contained CODH activity; its high rate of CO oxidation could protect hydrogenase from CO inhibition. Yet once partially purified from extracts, the CO-inducible hydrogenase in *Rx. gelatinosus* CBS is extremely sensitive to CO, with 50% inhibition observed at 3.9 µM dissolved CO [7]. The latter is consistent with the 5.8 to 40 µM *Ki* values of CO reported for most hydrogenases [59], [60]. This dramatic difference in tolerance to CO is likely attributed to the removal of the bulk of CODH from the partially purified *Rx. gelatinosus* CBS hydrogenase preparation used for the assay. While *Rs. rubrum*, *Rx. gelatinosus* strain CBS and strain S1 can grow in CO in darkness, with CO serving as a carbon substrate and energy source [4], [9], [61], an equal comparison of growth rates is complicated by the fact that all strains were grown under different conditions and with differing media components. No matter the growth rate, it is now well recognized that CO-inducible hydrogenase couples H_2_ evolution to proton translocation based on additional evidence including the effects of the electron transfer uncoupler carbonyl-cyanide *m*-chlorophenylhydrazone (CCCP) and the ATP synthesis inhibitor *N*, *N′*-dicyclohexylcarbodiimide (DCCD), with effects of the latter observed in both *Rs. rubrum* and *Rx. gelatinosus* CBS [9], [22].

## Conclusion

**Figure 4:**
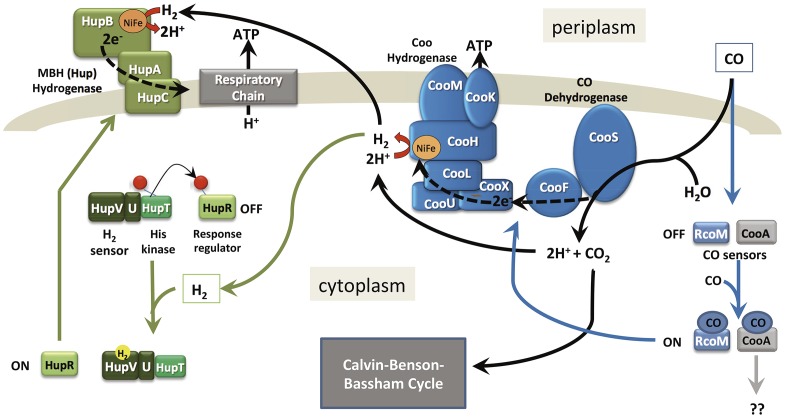
An overview of the CO and H_2_ signal transduction pathways and metabolism in *Rubrivivax gelatinosus* CBS.

The genomic information compiled in this report for *Rx. gelatinosus* CBS reveals features responsible for an autotrophic life style based on CO utilization. The *coo*, *hup* and *hyp* gene repertoire along with genes encoding the Calvin-Benson-Bassham (CBB) pathways (data not shown) enable growth using CO as the sole carbon substrate (Figure 4). This microbe contains a CO sensor, likely encoded by *rcoM*, which upon sensing CO initiates the transcription of the *coo* operon to enable CO oxidation and H_2_ production. This microbe also contains the H_2_-sensing hydrogenase HupUV, which is expected to work in concert with its cognate regulatory two-component system HupRT to regulate the expression of an H_2_-uptake hydrogenase HupAB. H_2_ oxidation ultimately provides electrons; that along with ATP generated from photosynthesis, participate in the CO_2_ fixation reaction via the CBB pathway leading to cell growth. *Rx. gelatinosus* CBS is therefore a model photosynthetic bacterium to study a network of intricate signal transduction pathways and the underlying regulations controlling the assimilation of one-carbon compounds such as CO and CO_2_.

## Supporting Information

Figure S1 The 242 kb contig from the *Rubrivivax gelatinosus* CBS *de novo* assembly is a distinct, circular genomic element. (a) Sequencing coverage from remapping SMRT sequencing reads onto the 242 kb contig from the *de novo* assembly. (b) Dot plot of the contig against itself, showing overlapping sequence at both ends (circled). (c) Dot plots between the 242 kb contig and the other two *de novo* assembly contigs, highlighting the absence of any sequence similarity between the 242 kb contig and the other contigs.
(TIF)

Figure S2 Curation of the bacterial chromosome assembly of *Rubrivivax gelatinosus* CBS. (a) Sequencing coverage from remapping SMRT sequencing reads onto the 4.7 Mb contig from the *de novo* assembly. The broad undulation in coverage is of biological origin due to the presence of more DNA in the sample near the origin of replication (*ori*) when cells are harvested in log growth phase. The large spike in coverage is highlighted (arrow). (b) Sequencing coverage over the 363 kb contig. (c) SMRTView zoom-in of the end of the 4.7 Mb contig showing the sequence read structure. Uniquely mapped reads are shown in gray, ambiguously mapping reads are highlighted in red. The dotplots show the end of the 4.7 Mb contig against itself (top), and the end of the 4.7 Mb contig against the beginning of the 362 kb contig (bottom).
(TIF)

Figure S3 *coo* operon comparison in the genome of *Rubrivivax gelatinosus* CBS strain and IL144. Genes flanking the upstream and downstream of the operon are present in both microbes, including a 48 bp region identical to the 3′ of *hypE* in *Rx. gelatinosus* CBS.
(TIF)

Figure S4 Methylome determination of *Rubrivivax gelatinosus* CBS. (a) Example section of the bacterial chromosome with kinetic signals indicating adenine methylation. (b) Scatter plot of kinetic scores over all 3 genomic elements. The threshold used for methyltransferase specificity determination is indicated by the dashed line. (c) Determined methyltransferase specificities. (d) Summary of detected methylated positions across the genome.
(TIF)

Figure S5 Kinetic score distributions for the identified methyltransferase specificities in *Rubrivivax gelatinosus* CBS.
(TIF)

Document S1 **Methylome.** The Methylome supplementary information describes the methodology used to determine the methylome in *Rx. gelatinosus* CBS. We uncover both type I and type II methyltransferases, both of which are N6-methyladenine. Further analysis involving cloning or knock outs of the methyltransferase genes would be necessary to determine the gene responsible for this specificity.
(DOCX)
