# Supplementary figures and images for: Genome Annotation Provides Insight into Carbon Monoxide and Hydrogen Metabolism in Rubrivivax gelatinosus
Source: PLoS One. 2014 Dec 5;9(12):e114551. doi: 10.1371/journal.pone.0114551 (PMC4257681; doi:10.1371/journal.pone.0114551)

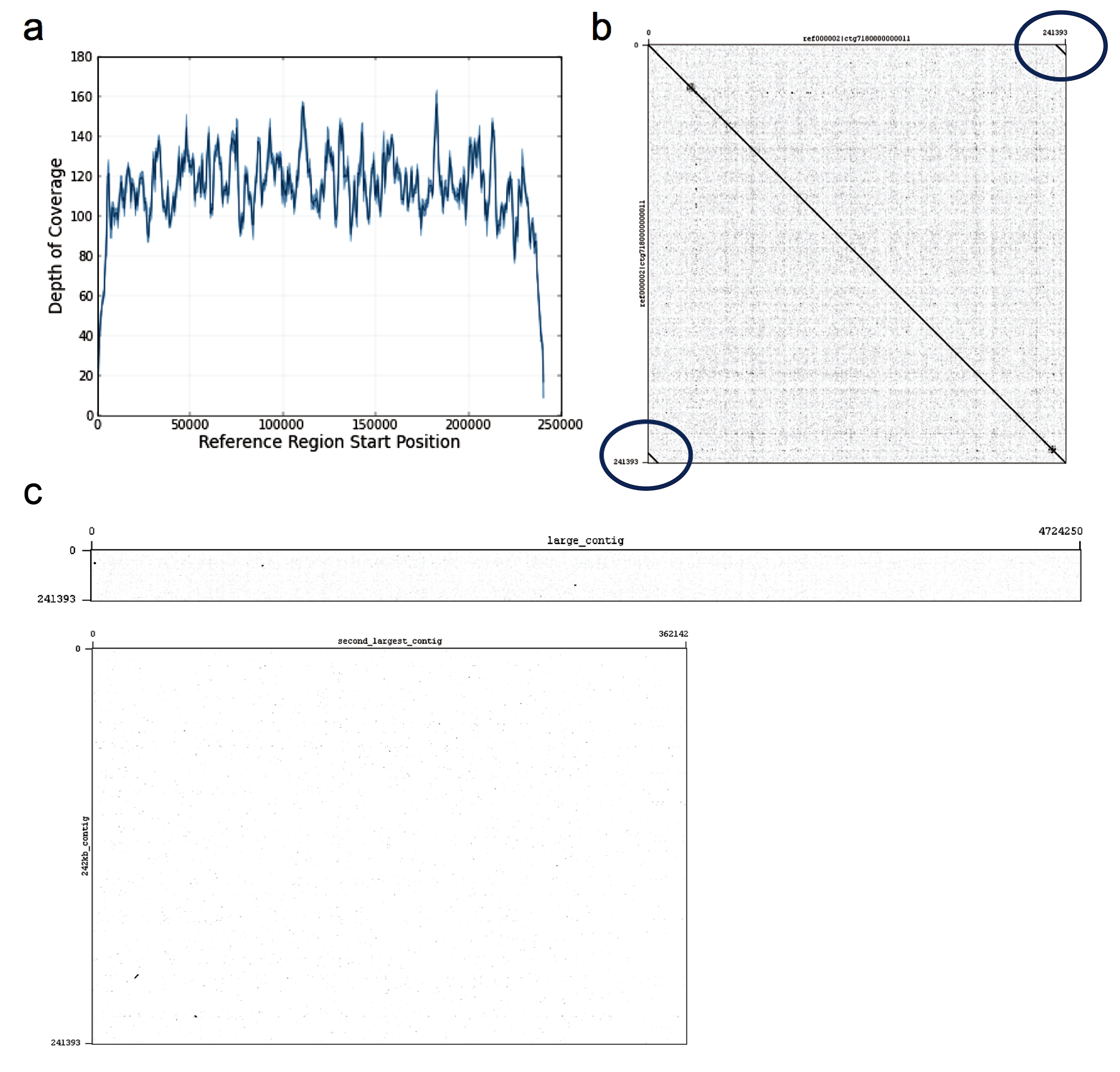

Supplement: Figure S1 — The 242 kb contig from the Rubrivivax gelatinosus CBS de novo assembly is a distinct, circular genomic element. (a) Sequencing coverage from remapping SMRT sequencing reads onto the 242 kb contig from the de novo assembly. (b) Dot plot of the contig against itself, showing overlapping sequence at both ends (circled). (c) Dot plots between the 242 kb contig and the other two de novo assembly contigs, highlighting the absence of any sequence similarity between the 242 kb contig and the other contigs. (TIF) [file pone.0114551.s001.tif]

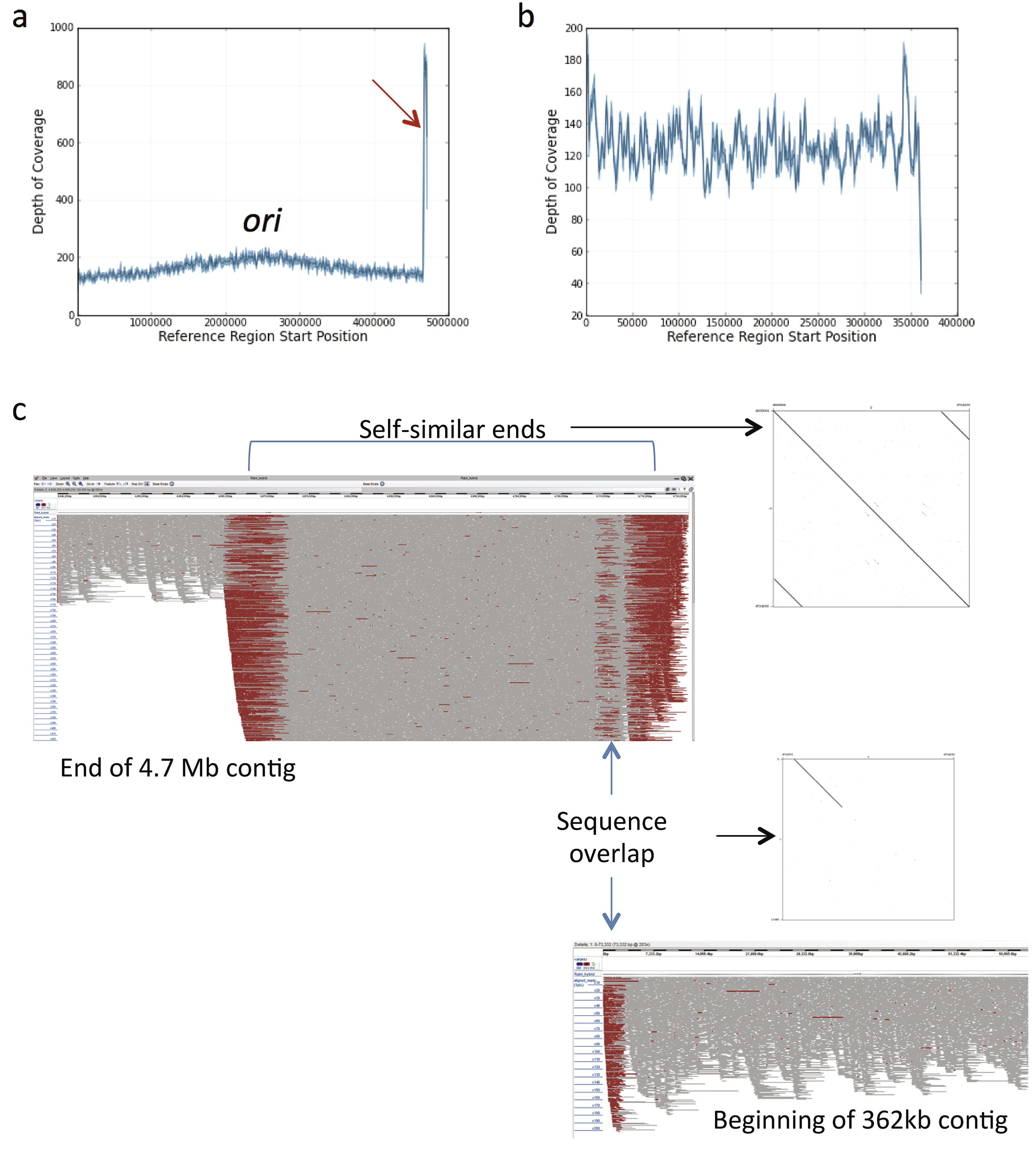

Supplement: Figure S2 — Curation of the bacterial chromosome assembly of Rubrivivax gelatinosus CBS. (a) Sequencing coverage from remapping SMRT sequencing reads onto the 4.7 Mb contig from the de novo assembly. The broad undulation in coverage is of biological origin due to the presence of more DNA in the sample near the origin of replication (ori) when cells are harvested in log growth phase. The large spike in coverage is highlighted (arrow). (b) Sequencing coverage over the 363 kb contig. (c) SMRTView zoom-in of the end of the 4.7 Mb contig showing the sequence read structure. Uniquely mapped reads are shown in gray, ambiguously mapping reads are highlighted in red. The dotplots show the end of the 4.7 Mb contig against itself (top), and the end of the 4.7 Mb contig against the beginning of the 362 kb contig (bottom). (TIF) [file pone.0114551.s002.tif]

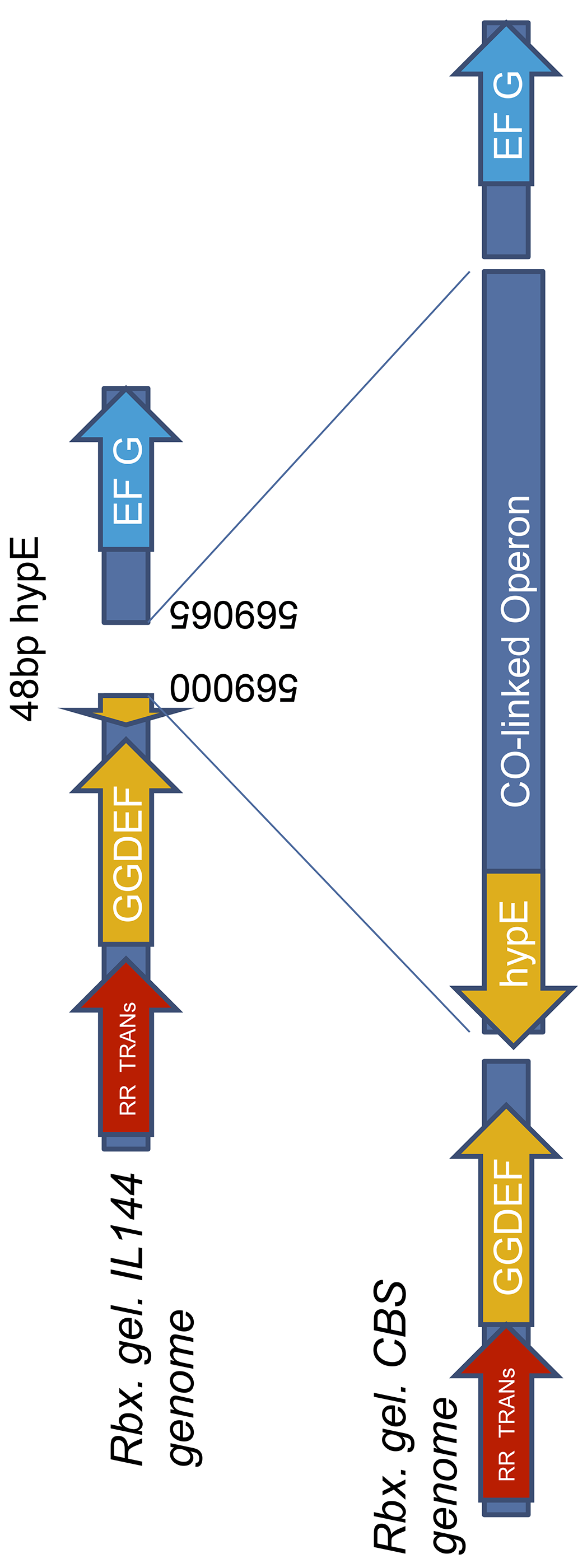

Supplement: Figure S3 — coo operon comparison in the genome of Rubrivivax gelatinosus CBS strain and IL144. Genes flanking the upstream and downstream of the operon are present in both microbes, including a 48 bp region identical to the 3′ of hypE in Rx. gelatinosus CBS. (TIF) [file pone.0114551.s003.tif]

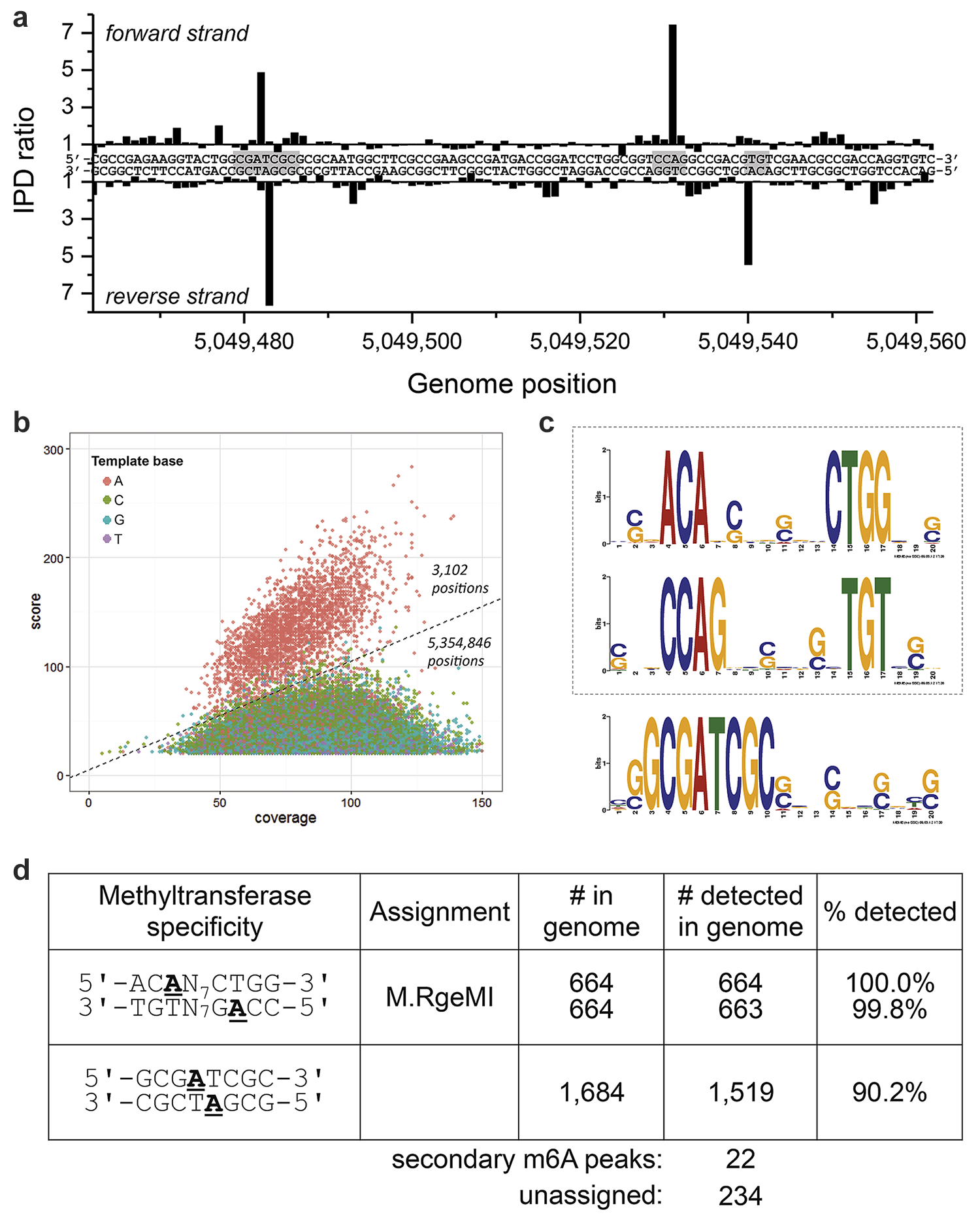

Supplement: Figure S4 — Methylome determination of Rubrivivax gelatinosus CBS. (a) Example section of the bacterial chromosome with kinetic signals indicating adenine methylation. (b) Scatter plot of kinetic scores over all 3 genomic elements. The threshold used for methyltransferase specificity determination is indicated by the dashed line. (c) Determined methyltransferase specificities. (d) Summary of detected methylated positions across the genome. (TIF) [file pone.0114551.s004.tif]

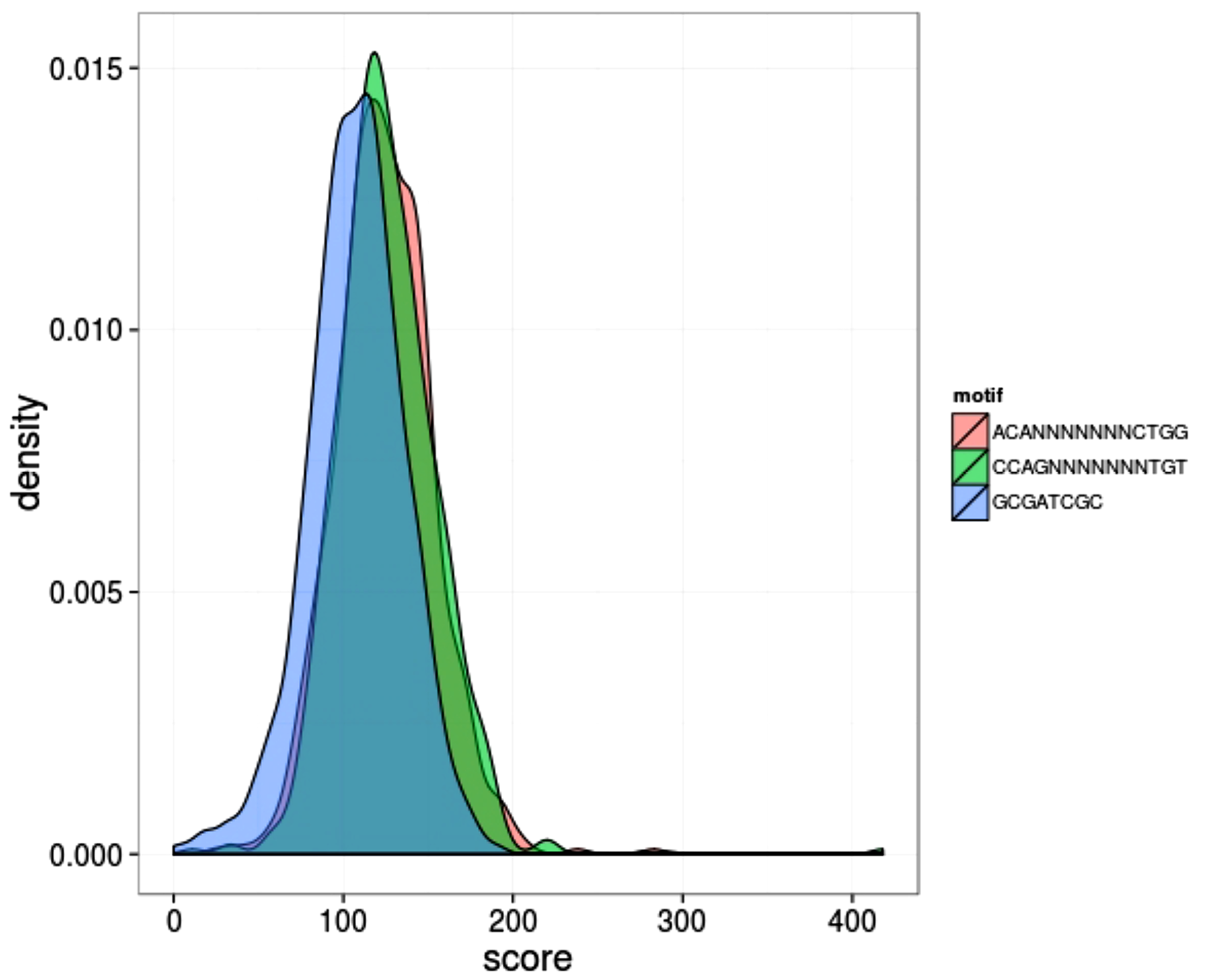

Supplement: Figure S5 — Kinetic score distributions for the identified methyltransferase specificities in Rubrivivax gelatinosus CBS. (TIF) [file pone.0114551.s005.tif]
