# Supplementary material for: Genome Annotation Provides Insight into Carbon Monoxide and Hydrogen Metabolism in Rubrivivax gelatinosus
Source: PLoS One. 2014 Dec 5;9(12):e114551. doi: 10.1371/journal.pone.0114551 (PMC4257681; doi:10.1371/journal.pone.0114551)
Supplement: Document S1 — Methylome. The Methylome supplementary information describes the methodology used to determine the methylome in Rx. gelatinosus CBS. We uncover both type I and type II methyltransferases, both of which are N6-methyladenine. Further analysis involving cloning or knock outs of the methyltransferase genes would be necessary to determine the gene responsible for this specificity. (DOCX) [file pone.0114551.s006.docx]

***Methylome.*** Methylome determination was performed as followed. Briefly, interpulse durations were measured as described previously and processed for all pulses aligned to each position in the reference sequence. To identify modified positions, we used Pacific Biosciences' SMRTPortal analysis platform, v. 1.3.1, which uses an *in silico* kinetic reference and a t-test based kinetic score detection of modified base positions (details available at <http://www.pacb.com/pdf/TN_Detecting_DNA_Base_Modifications.pdf>). Methylation signals, and assigned and predicted methyltransferase genes have been entered into REBASE.

***Rubrivivax gelatinosus* CBS Methylome.** The kinetic information collected during SMRT sequencing can be used to determine the epigenome of the bacterium. We determined two active methyltransferase specificities in this organism, both of which are N6-methyladenine (Supplementary Figure 4). The first is of type I with the recognition sequence of 5'-AC**^m6^A**N7CTGG-3' (with the underlined T representing A methylation on the opposite strand) and all occurrences in the genome were detected as methylated. Bioinformatic gene analysis produced one predicted type I restriction-modification (RM) system and the observed activity has been assigned to this system. The methyltransferase gene is located at positions 3,625,351 to 3,626,820 and has been designated as M.RgeI. A second, highly methylated m6A motif was identified, comprising the recognition sequence 5'-GCG**^m6^A**TCGC-3'. Around 90% of all genomic sites carrying this motif were detected as methylated with the thresholds chosen, and the kinetic signal strength in this context was somewhat lower than for the type I motif (Supplementary Figure 5). Bioinformatic predictions indicated the presence of six type II methyltransferases, all of which were orphan (i.e., no associated restriction enzyme gene) which could explain the lower degree of genome-wide methylation. Four of the six methyltransferase genes are predicted to constitute ^m6^A or ^m4^C methyltransferases and two are predicted ^m5^C methyltransferases (which were not expected to be detectable in this study because of the lower signal strength of m5C). Bioinformatic analysis could not assign a specific gene to the 5'-GCG**^m6^A**TCGC-3' activity, however it is most likely that either M.RgeMORFBP (144233-145999 on the big satellite element) or M.RgeMORFEP (3426111-3427097 on the chromosome) are responsible (R. Roberts, personal communication). Further analysis involving cloning or knock outs of the methyltransferase genes would be necessary to determine the gene responsible for this specificity. Several other predicted ^m6^A methyltransferase genes were determined to be inactive by this study due to the lack of methylated signals at the predicted sequence contexts.
